# Associations between body composition and autonomic cardiorespiratory modulation in young adults

**DOI:** 10.14814/phy2.70760

**Published:** 2026-02-09

**Authors:** Aurora Páramo‐Lira, Socorro Camarillo‐Romero, José de Jesús Garduño‐García, Pilar Cruz‐López, Eric Alonso Abarca‐Castro, Ana Karen Talavera‐Peña, José Javier Reyes‐Lagos

**Affiliations:** ^1^ School of Dentistry Autonomous University of the State of Mexico (UAEMéx) Toluca Mexico; ^2^ School of Chemistry Autonomous University of the State of Mexico (UAEMéx) Toluca Mexico; ^3^ Service of Internal Medicine Mexican Social Security Institute (IMSS) Metepec Mexico; ^4^ School of Medicine Autonomous University of the State of Mexico (UAEMéx) Toluca Mexico; ^5^ Department of Health Sciences Autonomous Metropolitan University Lerma Unit (UAM‐L) Lerma Mexico; ^6^ Department of Electrical Engineering, Bioelectronics Section Center for Research and Advanced Studies (Cinvestav) Mexico City Mexico

**Keywords:** autonomic nervous system, body composition, breathing rate variability, heart rate variability

## Abstract

This study investigated the associations between body composition and autonomic cardiac regulation, indexed by heart rate variability (HRV), in clinically healthy young adults, and additionally explored complementary breathing rate variability (BRV) and pulse–respiratory quotient (PRQ) indices. Ninety university students aged 18–23 years (35 men, 55 women) were evaluated. Linear and nonlinear HRV and BRV indices were derived from 5‐min R–R and breath‐to‐breath and PRQ time series. Body mass index (BMI) and bioelectrical impedance–based measures were obtained, including fat mass percentage (%FM), muscle mass, and visceral fat indices. HRV, BRV, PRQ, and composition indices were analyzed, and sex‐stratified correlations between body composition and autonomic indices were examined. Men showed greater respiratory variability and lower breathing rate than women. In men, BMI, %FM, and visceral fat indices correlated negatively with parasympathetic modulation and the complexity of cardiac and PRQ series, and positively with sympathetic modulation. In multivariable regression models adjusted for age, breathing rate, and physical activity (assessed with the IPAQ‐short form and expressed as total METs/week), higher %FM was independently associated with lower vagally mediated HRV indices in men, but not in women. Higher adiposity (higher BMI, %FM, and visceral fat indices), particularly in young men, was associated with reduced vagal HRV and diminished cardiorespiratory complexity.

## 1 INTRODUCTION

According to the World Health Organization (WHO), overweight and obesity are defined as abnormal or excessive fat accumulation, which are two of the principal risk factors that increase mortality and morbidity of different chronic diseases. An increase in overweight and obesity cases around the world has become a significant public health issue (World Health Organization, 2021). In Mexico, obesity has been declared a public health emergency since 2016, primarily due to its 1.3 percentage point annual increase since 2006. As of 2023, 38.9% of the population (aged 20 and older) had obesity, and 37.3% were overweight (Shamah‐Levy et al., 2024). Furthermore, cardiovascular diseases are a leading cause of death and can affect people from a young age. A recent American Heart Association scientific statement on cardiovascular health in the transition from adolescence to emerging adulthood underscores that the period from roughly 18 to the mid‐20s is a critical window in which many risk factors are already present and long‐term trajectories of cardiovascular health are established. This highlights the importance of studying emerging adults such as the young university students included in this study (Scott et al., 2025).

The most common method for classifying overweight or obesity is body mass index (BMI) (World Health Organization, 2021), although it does not account for all components of body composition. A technique used to estimate body composition is bioelectrical impedance analysis (BIA), which determines the fat mass, fat‐free mass, visceral mass, and total body water (Mulasi et al., 2015). In this way, diverse meta‐analyses have concluded that having a normal BMI but a high‐fat percentage (normal weight obesity (NWO)) increases the risk of cardiometabolic risk factors (Mohammadian Khonsari et al., 2022; Rakhmat et al., 2022). In older adults, sarcopenic obesity—characterized by an increase in fat mass and a decrease in muscular mass (Rakhmat et al., 2022)—has been associated with a higher risk of metabolic disorders, morbidity, cardiovascular diseases, and mortality (Binu et al., 2023; Zhang et al., 2019). These observations suggest that calculating BMI alone may be insufficient to determine the risk of obesity‐related complications.

The autonomic nervous system (ANS) regulates cardiac and respiratory functioning in normal physiologic conditions (Karemaker, 2017; Wu & Vaseghi, 2020). Thus, disorders such as obesity or overweight represent a health risk factor for people and may modify the ANS modulation. A noninvasive way of analyzing its modulation and balance (Catai et al., 2020) is by obtaining the heart rate variability (HRV) and the breathing rate variability (BRV) (Soni & Muniyandi, 2019).

The HRV traces the oscillations between the RR intervals or the oscillations between consecutive instantaneous heart rates, while the BRV determines the intervals between breath to breath (BB). In this way, higher HRV values are generally associated, at the group level, with better cardiovascular adaptability and more efficient autonomic regulation. In contrast, lower HRV indices have been linked in epidemiological studies to increased risk of several diseases (Shaffer et al., 2014; Vanderlei et al., 2009) and all‐cause mortality (Fang et al., 2020). According to relevant research by Cvijetic et al. (2023), male young participants with higher fat mass and lower muscle mass had lower vagally mediated HRV indices, consistent with reduced parasympathetic modulation.

Likewise, it is important to consider that breathing rate affects HRV, because inhalation causes heart rate to increase and blood pressure (BP) to decrease, while exhalation causes HR to decrease and increases BP (Karemaker, 2017; Shaffer & Meehan, 2020). This is known as respiratory sinus arrhythmia and can also be used to study vagal‐cardiac nerve modulation (Katona & Jih, 1975). However, the Pulse Respiration Quotient (PRQ) captures the complex interplay between the cardiac and respiratory systems, allowing us to evaluate the subject‐specific physiological or pathophysiological state in general (Scholkmann & Wolf, 2019).

Sex differences are relevant when examining links between body composition and autonomic cardiorespiratory regulation. First, men and women show consistent differences in baseline autonomic balance and HRV profiles: a meta‐analysis of 63,612 individuals found that females exhibited higher vagally‐mediated HRV (higher high‐frequency power, lower LF/HF ratio) despite a higher resting heart rate (shorter RR interval) than males (Koenig & Thayer, 2016). Moreover, sex‐specific body‐composition characteristics (for example, higher fat‐mass percentage in women and greater muscle mass/greater visceral‐fat burden in men) may translate into different autonomic consequences; for instance, cross‐sectional work in young adults reported associations between body fat and HRV (Köchli et al., 2020). Finally, respiratory sexual dimorphism—women typically have smaller absolute lung volumes and vital capacity compared with men (even after size adjustment), and often greater breathing frequency—can modify the magnitude of respiratory sinus arrhythmia and thus potentially alter HRV–composition relationships (LoMauro & Aliverti, 2018). Together, these physiological considerations provide a clear rationale for exploring sex‐stratified associations in the present study.

Entropic measurements based on physiological signals, such as heart rate and breathing rate fluctuations, can also be a valuable tool for detecting pathology because they capture nonlinear dynamics, which help evaluate irregularities and/or the complexity of a biological system (Chen et al., 2017; Siciliano, 2017). In this context, biodynamic data can offer early insights into the progression or worsening of a disease. A well‐balanced biological system, which is healthy, reduces energy and increases entropy. Conversely, an unhealthy organism or one that has experienced a decrease in complexity faces greater challenges in adaptation (Siciliano, 2017).

The literature indicates that body composition can influence autonomic modulation by reducing global HRV (Monteze et al., 2015) and parasympathetic modulation (Chang et al., 2021), while increasing sympathetic modulation, which in turn increases the risk of cardiovascular morbidity and mortality (Yadav et al., 2017). However, there is limited evidence on the specific interactions between detailed body composition metrics and cardiorespiratory autonomic modulation. In addition, Latino adults, including Mexican young adults, are underrepresented in studies that jointly examine HRV, body composition, and health behaviors. Understanding these interactions in a clinically healthy Mexican young‐adult population could provide insights into early preventive measures for cardiovascular health.

This study primarily aimed to examine the associations between specific body composition metrics—body mass index (BMI), fat mass percentage (%FM), muscle mass (kgM), and visceral fat indices (visceral fat rate [VFR] and normalized visceral fat rate [nVFR])—and autonomic cardiac regulation, as indexed by HRV, in clinically healthy young adults. As a secondary, exploratory aim, we also characterized complementary BRV and PRQ indices as additional descriptors of autonomic cardiorespiratory modulation. We hypothesized that higher fat mass and visceral fat, as well as muscle mass, would be significantly correlated with autonomic indices reflecting variations in parasympathetic and sympathetic modulation, and that these associations would differ between men and women. By analyzing these relationships in a predominantly nonobese, low‐risk sample, we aimed to refine current understanding of how established body‐composition markers relate to early autonomic cardiorespiratory regulation and to evaluate whether subtle, sex‐dependent patterns are already detectable at young ages.

## 2 MATERIALS AND METHODS

### 2.1 Study design and participants

An observational cross‐sectional study was conducted from March 28, 2023, to March 7, 2024, among young Mexican university students aged between 18 and 23. Participants were recruited using a non‐probabilistic convenience sample of undergraduate students enrolled at the Autonomous University of the State of Mexico (UAEMéx). Race/ethnicity (including Latino identity) was not formally assessed; participants were recruited from a university population in Mexico. Each potential participant was informed about the study, its objectives, and the procedures involved and was invited to participate. Those who agreed provided their authorization by signing a written informed consent form. The inclusion criteria were male or female students enrolled in bachelor's degree programs at the UAEMéx who were clinically healthy and willing to provide informed consent.

The exclusion criteria included participants diagnosed with pathologies such as cardiovascular diseases, respiratory disorders, diabetes, or any other chronic illnesses that could affect HRV or BRV. Participants who consumed medications that could alter HRV or BRV, as well as those who had consumed coffee, chocolate, energy drinks, smoked, or exercised within 12 h before the study, were excluded. All measurements were scheduled in the morning under standardized resting conditions; however, fasting status, alcohol abstinence, and water intake were not formally standardized or recorded. Additionally, individuals with incomplete data or those who refused to sign the informed consent were not included in the analysis.

The elimination criteria involved removing data from participants for whom it was not possible to detect R waves in the electrocardiogram signals or B peaks in the respiratory signals. Additionally, participants who did not complete the study were also discarded.

The study was conducted in accordance with the ethical principles outlined in the Helsinki Declaration (Asociación Medica Mundial, 2013), safeguarding each participant's rights, interests, and dignity. All participants signed an informed consent form before participating, and all methods were performed in accordance with relevant guidelines and regulations. This research was approved by the Research Ethical Committee of the School of Medicine of the Autonomous University of the State of Mexico, with registry number 005.2023.

### 2.2 Physical activity assessment and anthropometric measurements

Physical activity and anthropometric measurements were comprehensively assessed in all volunteers who participated in the study. Personal data were collected using a structured face‐to‐face questionnaire, including family history of type 2 diabetes and arterial hypertension in first‐degree relatives. Physical activity levels were assessed using the International Physical Activity Questionnaire (IPAQ) short form (Medina et al., 2013). This tool categorizes physical activity levels as low, moderate, or high (vigorous) based on Metabolic Equivalent of Task (MET) scores.

Height was measured using a Seca 213 stadiometer (Serial number 10000000717029). Weight, fat mass, and muscle mass were assessed using bioelectrical impedance analysis (Tanita BC‐533, Tokyo, Japan). During these measurements, participants wore minimal clothing and were barefoot, with electrodes placed on their toes and heels, and their arms hanging slightly away from their bodies. Blood pressure was measured using a manually calibrated sphygmomanometer and a stethoscope.

Body mass index (BMI) was calculated as weight (kg) divided by height squared (m^2^), and participants were classified according to the World Health Organization (WHO) standards (Weir & Jan, 2022; WHO, 2010) into underweight, normal weight, overweight, and obese categories. Body fat percentage was evaluated according to the criteria of Gallagher et al. (2000), which categorized participants as having low, normal, high, or obese body fat. Additionally, the relative muscle mass (RMM) was determined by dividing the muscle mass (kgM) by the body mass (kg) × 100 (Hong et al., 2017), and the normalized visceral fat rate (nVFR) was determined by dividing the visceral fat rate (VFR) obtained through bioelectrical impedance by the participant's weight (kg) (Escutia‐Reyes et al., 2021).

### 2.3 Electrocardiographic and respiratory recordings

Electrocardiographic (ECG) and respiratory measurements were performed in accordance with the guidelines established by the European Society of Cardiology (ESC) and the North American Society of Pacing and Electrophysiology (NASPE) (Camm et al., 1996).

The ECG and respiratory signals were simultaneously recorded in a controlled environment between 7:30 and 11:00 am to minimize diurnal variations. All measurements were conducted in a quiet room to reduce external noise and distractions. Each participant's physiological signals were captured over a 5‐min period using a BIOPAC MP150 system (BIOPAC Systems, Inc., Goleta, CA, USA) equipped with ECG100C and RESP100 amplification modules. The sampling frequency was set to 1000 Hz to ensure high‐resolution data acquisition.

ECG recordings were obtained in a lead II configuration using disposable electrodes coated with electrolytic gel. The skin area where the electrodes were placed was meticulously cleaned with methyl alcohol to ensure optimal signal quality. For respiratory measurements, a respiratory effort band was placed around the participant's waist to capture thoracic excursions during breathing.

All recordings were conducted with participants in a supine position to standardize physiological conditions and minimize postural influences on cardiovascular and respiratory parameters. During the recording sessions, participants were instructed to remain still, maintain spontaneous breathing patterns, and keep their eyes open. They were also required to remove any mobile devices or metal objects to prevent signal interference. Participants were ensured to be in a physiologically stable state, relaxed, and fully awake throughout the data acquisition process.

### 2.4 Processing electrocardiographic and respiratory signals

ECG signals were exported as text files using AcqKnowledge Software (BIOPAC Systems, Inc., USA). Interbeat RR intervals were computed from the ECGs by using the QRStool software (Allen et al., 2011), which allowed visual inspection and manual correction of R‐peak detection and ectopic beats. Linear (time‐ and spectral‐domain) and nonlinear analyses of the RR interval data were performed using Kubios HRV Standard Software (Tarvainen et al., 2014) after importing the artifact‐corrected RR interval series obtained from QRStool. The cardiac analysis included both linear and non‐linear indices such as the parasympathetic nervous system index (PNS index) (in Kubios, a composite marker of vagal modulation computed from mean RR, RMSSD, and normalized SD1, standardized to normative values; higher values indicate higher parasympathetic modulation) (Kubios HRV Scientific, 2025), sympathetic nervous system index (SNS index), stress index, standard deviation of RR intervals (SDRR, ms), mean heart rate (HR mean, beats/min), root mean square of successive RR intervals differences (RMSSD, ms), the percentage of successive RR intervals that differ by more than 50 ms (pNN50, %), low‐frequency power (LF, normalized units), high‐frequency power (HF, normalized units), approximate entropy (ApEn), and sample entropy (SampEn).

Respiratory signals were analyzed using MATLAB (The MathWorks, Inc., Natick, Massachusetts, USA). The temporal positions of the respiratory peaks were detected using custom in‐house scripts in MATLAB, facilitating the calculation of the breath‐to‐breath BB signal. Consequently, diverse temporal BRV indices such as the mean respiratory rate (BR), the standard deviation of the BB intervals (SDBB, ms), and the root mean square of successive BB interval differences (RMSSD_BB_, ms) were computed. The pulse‐respiratory quotient (PRQ) time series were calculated from RR and BB values, as described in the literature (Scholkmann & Wolf, 2019). These values were further processed using the PhyBios program (Silva et al., 2020) to obtain the mean PRQ (mPRQ), the sample entropy (SampEn_PRQ_), and the fuzzy entropy (FuzzEn_PRQ_) for each PRQ signal.

### 2.5 Statistical analysis

Continuous variables were summarized as means and standard deviations (SD), while categorical variables were expressed as frequencies and percentages. Differences between categorical variables were assessed using the chi‐square test. Normality was assessed with the Kolmogorov–Smirnov test; for quantitative variables, the Student's *t*‐test or the Mann–Whitney *U* test was used, depending on the distribution. For all sex‐based comparisons, we additionally computed effect sizes (Cramér’s V for chi‐square tests, Hedges' g for *t*‐tests and Cliff's δ for Mann–Whitney *U* tests) to quantify the magnitude of group differences. Bivariate correlations were used to examine associations between cardiorespiratory indices and body‐composition measures, using Pearson's r for parametric data and Spearman's rho for nonparametric data. The significance level of *p* < 0.05 was used for these tests.

In addition to the bivariate correlations, we fitted multiple robust linear regression models with standardized variables to examine whether the association between %FM and primary vagally mediated HRV indices (RMSSD, HFnu, and PNS index) remained after adjustment for sex, age, breathing rate, and METs. We first estimated pooled models including sex as a covariate, and then sex‐stratified models; for RMSSD and the PNS index, we additionally tested a %FM × sex interaction in separate pooled models to formally assess sex‐specific slopes. The false discovery rate across these primary HRV models was controlled using the Benjamini–Hochberg procedure (*q* < 0.05). Conventional analyses were performed in SPSS v26 (IBM Corp., Armonk, NY, USA), and regression modeling and figures were created in MATLAB R2024a (MathWorks, Natick, MA, USA).

## 3 RESULTS

Out of the initial cohort of 123 university students, five participants were excluded due to age constraints, 20 due to the presence of pathologies affecting HRV, one due to the consumption of medications influencing HRV, six for not meeting the required physiological conditions for electrocardiographic measurements, and one due to cardiorespiratory signals exceeding the normal range. Consequently, the data from 90 university students were analyzed, comprising 35 men (mean age 19.4 ± 1.3 years) and 55 women (mean age 20.0 ± 1.4 years). The sample included more women than men due to volunteer availability during this convenience‐based recruitment; therefore, all primary analyses were performed sex‐stratified.

The demographic and clinical characteristics of the sample by sex are summarized in Table 1. No statistically significant sex differences were detected in age, smoking status, or family history of diabetes or hypertension (effect sizes are reported in Table 1). Men reported higher levels of physical activity and were more frequently classified in the vigorous activity category. Women showed slightly higher resting oxygen saturation, whereas men had higher systolic and diastolic blood pressure.

**TABLE 1:** Demographic and clinical characteristics of young individuals by sex (mean ± SD or *n* (%)).

Variable	Total (*N* = 90)	Men (*n* = 35)	Women (*n* = 55)	*p*‐value	Effect size
Personal history					
Age, years^‡^	19.83 ± 1.47	19.49 ± 1.38	20 ± 1.40	0.072	−0.22 (δ)
Current smoker^§^	20 (22.2%)	10 (28.6%)	10 (18.2%)	0.250	0.12 (V)
Physical activity, METS/week^‡^	2525.71 ± 2112.02	3323.98 ± 2321.35	2017.67 ± 1811.94	0.006	0.34 (δ)
Physical activity classification^§^					
Low physical activity	17 (18.9%)	4 (11.4%)	13 (23.6%)	0.042*	0.27 (V)
Moderate physical activity	36 (40.0%)	11 (31.4%)	25 (45.5%)		
Vigorous physical activity	37 (41.1%)	20 (57.1%)	17 (30.9%)		
Family inheritance history					
Family history of type 2 diabetes^§^	28 (31.1%)	8 (22.9%)	20 (36.4%)	0.180	0.14 (V)
Family history of arterial hypertension^§^	47 (52.2%)	19 (54.3%)	28 (50.9%)	0.756	0.03 (V)
Vital signs					
SPO_2_ ^‡^	93.74 ± 2.64	93.17 ± 1.96	94.11 ± 2.95	0.014*	−0.31 (δ)
Systolic pressure, mmHg^‡^	106.52 ± 10.86	112.23 ± 11.18	102.89 ± 9.01	0.001*	0.46 (δ)
Diastolic pressure, mmHg^‡^	73.03 ± 9.09	76.26 ± 9.79	70.98 ± 8.05	0.016*	0.30 (δ)
Hours of sleep (before the study)^‡^	6.56 ± 1.32	6.71 ± 1.12	6.46 ± 1.44	0.178	0.16 (δ)
Body composition					
Weight (kg)^†^	63.63 ± 10.43	69.60 ± 9.98	59.84 ± 8.88	0.001*	1.04 (g)
Height (m)^†^	162.77 ± 7.89	169.63 ± 5.66	158.40 ± 5.71	0.001*	1.95 (g)
Fat mass (%) (%FM)^†^	25.31 ± 8.05	18.79 ± 5.97	29.46 ± 6.28	0.001*	−1.72 (g)
Muscle (kg) (kgM)^‡^	44.92 ± 7.79	53.31 ± 5.22	39.57 ± 2.85	0.001*	0.99 (δ)
Relative muscle mass (%) (RMM)^‡^	70.94 ± 7.76	77.22 ± 5.92	66.95 ± 5.95	0.001*	0.78 (δ)
Visceral fat rate (VFR)^‡^	2.71 ± 2.05	3.37 ± 2.57	2.29 ± 1.51	0.052	0.24 (δ)
Normalized visceral fat rate (nVFR)^‡^	0.03 ± 0.02	0.04 ± 0.02	0.03 ± 0.01	0.513	0.09 (δ)
Body mass index (BMI)^†^	23.92 ± 3.02	24.11 ± 2.88	23.79 ± 3.13	0.621	0.10 (g)
BMI classification^§^					
Underweight	2 (2.2%)	0 (0.0%)	2 (3.6%)	0.416	0.18 (V)
Healthy weight	54 (60.0%)	21 (60.0%)	33 (60%)		
Overweight	33 (36.7%)	13 (37.1%)	20 (36.4%)		
Obesity class I	1 (1.1%)	1 (2.9%)	0 (0.0%)		
Body fat % category^§^					
Low fat	9 (10%)	3 (8.6%)	6 (10.9%)	0.951	0.06 (V)
Healthy	53 (58.9%)	20 (57.1%)	33 (60.0%)		
High fat	19 (21.1%)	8 (22.9%)	11 (20.0%)		
Obesity	9 (10.0%)	4 (11.4%)	5 (9.1%)		

*Note*: Effect sizes are Hedges g for †, Cliff's δ for ‡, and Cramér's V for §. Positive effect sizes indicate higher values in men. Blood pressure was measured in the seated position after 5 min of rest.

* The difference in means is significant at the *p* < 0.05 level.

^†^ Student's *t*‐test.

^‡^ Mann–Whitney *U* test.

^§^ Chi‐square test.

Regarding body composition, men were taller and heavier, with greater absolute and relative muscle mass and lower body fat percentage than women. At the same time, BMI and the distribution of BMI and %FM categories were broadly comparable between sexes. Men also tended to have higher visceral fat indices.

**FIGURE 1:**
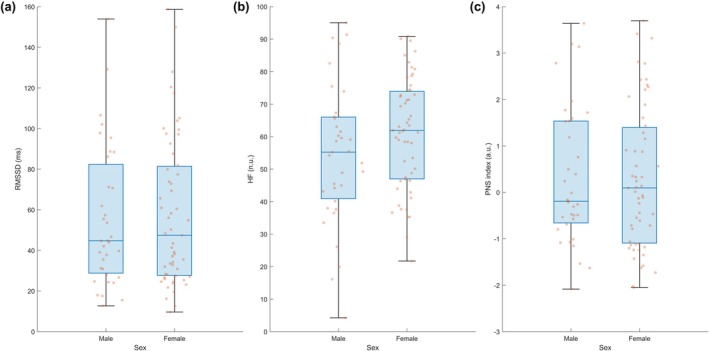
Distribution of vagally mediated HRV indices by sex. Box‐and‐jitter plots of (a) RMSSD (ms), (b) high‐frequency power in normalized units (HF, n.u.), and (c) parasympathetic nervous system (PNS) index (a.u.) in male and female university students (*n* = 90). No significant between‐sex differences were observed for RMSSD (*p* = 0.885), HF (*p* = 0.120), or PNS index (*p* = 0.937). Boxes represent the interquartile range with the median as the horizontal line; whiskers indicate the full data range; jittered dots correspond to individual participants.

As shown in Table 2, no statistically significant sex differences were detected in most HRV and PRQ indices; effect sizes are reported in Table 2. In contrast, respiratory measures showed apparent sex‐related differences: men exhibited a lower breathing rate, but higher breath‐to‐breath variability (SDBB and RMSSD_BB_) compared with women. These patterns are visually consistent with the group statistics and are presented in Figure S1. The distributions of the primary vagally mediated HRV indices by sex are shown in Figure 1.

**TABLE 2:** Linear and nonlinear indices of HRV, BRV, and PRQ (mean ± SD), by sex.

Parameter	Men (mean ± SD)	Women (mean ± SD)	*p*‐value	Effect size (g or δ)
PNS index^‡^	0.31 ± 1.47	0.30 ± 1.53	0.937	0.01 (δ)
SNS index^‡^	0.26 ± 1.30	0.48 ± 1.53	0.646	−0.15 (g)
Stress index^‡^	10.34 ± 4.86	11.32 ± 5.65	0.560	−0.07 (δ)
SDRR (ms)^‡^	50.95 ± 23.49	47.68 ± 24.85	0.369	0.11 (δ)
HR Mean (bpm)^†^	68.40 ± 10.76	70.17 ± 10.46	0.443	−0.06 (δ)
RMSSD (ms)^‡^	55.60 ± 34.61	57.78 ± 36.49	0.885	−0.02 (δ)
pNN50 (%)^‡^	29.17 ± 23.57	31.72 ± 25.49	0.706	−0.05 (δ)
LF (n.u)^†^	45.56 ± 21.48	38.74 ± 17.04	0.118	0.36 (g)
HF (n.u)^†^	54.38 ± 21.49	61.15 ± 17.07	0.120	−0.36 (g)
ApEn^†^	1.09 ± 0.08	1.12 ± 0.09	0.086	−0.37 (g)
SampEn^†^	1.67 ± 0.27	1.76 ± 0.24	0.130	−0.34 (g)
BR (breaths/min)^†^	15.65 ± 4.45	17.88 ± 3.40	0.014*	−0.58 (g)
SDBB (ms)^‡^	911.20 ± 548.19	583.15 ± 300.94	0.002*	0.38 (δ)
RMSSD_BB_ (ms)^‡^	1026.94 ± 597.45	695.06 ± 374.55	0.006*	0.34 (δ)
mPRQ^‡^	4.93 ± 1.87	4.34 ± 1.40	0.128	0.19 (δ)
SampEn_PRQ_ ^†^	1.24 ± 0.44	1.42 ± 0.40	0.069	−0.40 (g)
FuzzEn_PRQ_ ^†^	1.08 ± 0.40	1.22 ± 0.37	0.133	−0.33 (g)

*Note*: Effect size: Hedges' g for *t*‐tests and Cliff's δ for Mann–Whitney *U* tests.

Abbreviations: ApEn, approximate entropy; BR, breathing rate; FuzzEn_PRQ,_ fuzzy entropy of PRQ; HF, high frequency; HR, heart rate; LF, low frequency; mPRQ, mean pulse‐respiration quotient; pNN50, percentage of successive RR intervals that differ by more than 50 ms; PNS index, parasympathetic nervous system index; RMSSD, root mean square of successive RR interval differences; RMSSD_BB_, root mean square of successive breath‐to‐breath interval differences; SampEn, sample entropy; SampEn_PRQ_, sample entropy of PRQ; SDBB, standard deviation of breath‐to‐breath intervals; SDRR, standard deviation of RR intervals; SNS index, sympathetic nervous system index.

* The difference in means is significant at the *p* < 0.05 level.

^†^ Student's *t*‐test.

^‡^ Mann–Whitney *U* test.

The linear correlations between HRV measures and body composition parameters among university students by sex are presented in Table 3. Significant correlations were observed among various indices for men and women.

**TABLE 3:** Linear correlations between HRV measures and body composition parameters among young individuals by sex.

	PNS index^†^	SNS index^†^	Stress index^†^	SDRR^†^	Mean HR^‡^	RMSSD^†^	pNN50^†^	Lf^‡^	HF^‡^	ApEn^‡^	SampEn^‡^
Men											
BMI	−0.383	0.305	0.263	−0.260	0.319	−0.384	−0.491	0.482	−0.481	−0.068	−0.571
	0.023*	0.074	0.128	0.132	0.061	0.023*	0.003*	0.003*	0.003*	0.699	0.001*
%FM	−0.455	0.388	0.38	−0.361	0.426	−0.437	−0.507	0.384	−0.384	−0.024	−0.474
	0.006*	0.021*	0.024*	0.033*	0.011*	0.009*	0.002*	0.023*	0.023*	0.891	0.004*
kgM	−0.177	0.117	0.116	−0.114	−0.055	−0.278	−0.403	0.644	−0.644	−0.126	−0.381
	0.308	0.505	0.507	0.513	0.755	0.106	0.016*	0.001*	0.001*	0.470	0.024*
RMM	0.320	−0.339	−0.200	0.202	−0.423	0.203	0.232	0.102	−0.102	0.045	0.316
	0.061	0.047*	0.249	0.244	0.011*	0.242	0.180	0.560	0.560	0.796	0.064
VFR	−0.481	0.405	0.398	−0.379	0.424	−0.487	−0.569	0.435	−0.434	−0.037	−0.498
	0.003*	0.016*	0.018*	0.025*	0.011*	0.003*	0.001*	0.009*	0.009*	0.832	0.002*
nVFR	−0.485	0.421	0.399	−0.384	0.438	−0.467	−0.538	0.375	−0.375	0.018	−0.448
	0.003*	0.012*	0.017*	0.023*	0.008*	0.005*	0.001*	0.026*	0.027*	0.919	0.007*
Women											
BMI	0.146	−0.070	−0.074	0.102	−0.004	0.163	0.109	−0.181	0.181	−0.066	−0.076
	0.287	0.612	0.589	0.457	0.980	0.235	0.428	0.186	0.186	0.632	0.580
%FM	0.113	−0.031	−0.046	0.067	0.009	0.118	0.048	−0.182	0.182	−0.141	−0.172
	0.410	0.825	0.736	0.629	0.946	0.393	0.727	0.184	0.183	0.303	0.210
kgM	0.228	−0.178	−0.159	0.168	−0.155	0.220	0.192	−0.281	0.281	−0.169	−0.076
	0.094	0.193	0.245	0.220	0.258	0.106	0.160	0.038*	0.038*	0.218	0.582
RMM	−0.026	−0.077	−0.070	−0.045	−0.074	−0.090	−0.023	0.121	−0.119	−0.011	0.000
	0.851	0.578	0.609	0.744	0.591	0.515	0.867	0.378	0.386	0.935	0.999
VFR	0.083	0.000	−0.005	0.032	0.072	0.072	0.007	−0.031	0.031	−0.041	−0.217
	0.547	1.000	0.971	0.816	0.603	0.600	0.959	0.820	0.82	0.766	0.111
nVFR	−0.001	0.080	0.074	−0.041	0.076	−0.022	−0.089	−0.003	0.003	−0.034	−0.208
	0.992	0.562	0.592	0.768	0.579	0.871	0.518	0.982	0.982	0.807	0.128

Abbreviations: %FM, fat mass percentage; ApEn, approximate entropy; BMI, body mass index; HF, high‐frequency; HR Mean, heart rate mean; kgM, muscle kilograms; LF, low‐frequency; nVFR, normalized visceral fat rate; pNN50, percentage of successive RR intervals that differ by more than 50 ms; PNS index, parasympathetic nervous system index; RMM, relative muscle mass; RMSSD, root mean square of successive RR interval differences; SampEn, sample entropy; SDRR, standard deviation of RR intervals; SNS index, sympathetic nervous system index; VFR, visceral fat rate.

* *p* < 0.05 = Correlation significance level obtained with † Spearman's rho and ‡ Pearson's r.

In university men, BMI showed several moderate negative correlations with HRV indices, including PNS, RMSSD, pNN50, HF, and SampEn. Additionally, BMI had a mild positive correlation with LF.

In university men, kgM showed moderate negative associations with pNN50, HF, and SampEn, and a strong positive association with LF. Additionally, RMM showed moderate negative correlations with the SNS index and HR mean.

%FM, VFR, and nVFR in university men demonstrated moderate negative associations with several HRV indices, including PNS, SDRR, RMSSD, pNN50, HF, and SampEn. Conversely, these body composition parameters had moderate positive associations with SNS, stress index, HR Mean, and LF.

In university women, kgM showed a moderate negative association with LF and a moderate positive association with HF.

These findings highlight significant correlations between body composition parameters and HRV measures, highlighting differences between the sexes among university students. For detailed numerical values and exact correlation coefficients, refer to Table 3.

The linear correlations between BRV‐PRQ measures and body composition parameters among university students by sex are presented in Table 4. Significant correlations were observed among indices for men, but none for women.

**TABLE 4:** Linear correlations between BRV‐PRQ measures and body composition parameters among young individuals by sex.

	BR^‡^	SDBB^†^	RMSSD_BB_^†^	mPRQ^†^	SampEn_PRQ_^‡^	FuzzEn_PRQ_^‡^
Men						
BMI	−0.238	0.471	0.385	0.373	−0.499	−0.519
	0.169	0.004*	0.022*	0.027*	0.002*	0.001*
%FM	−0.188	0.371	0.32	0.309	−0.517	−0.539
	0.278	0.028*	0.061	0.071	0.001*	0.001*
KgM	−0.327	0.395	0.352	0.284	−0.253	−0.277
	0.055	0.019*	0.038*	0.098	0.143	0.107
RMM	0.019	−0.021	−0.054	−0.264	0.282	0.307
	0.914	0.906	0.757	0.126	0.100	0.073
VFR	−0.257	0.426	0.357	0.385	−0.514	−0.544
	0.136	0.011*	0.035*	0.022*	0.002*	0.001*
nVFR	−0.21	0.379	0.303	0.384	−0.514	−0.545
	0.226	0.025*	0.077	0.023*	0.002*	0.001*
Women						
BMI	0.205	−0.137	−0.128	−0.156	0.017	0.021
	0.134	0.324	0.350	0.255	0.902	0.882
%FM	0.128	−0.097	−0.135	−0.078	0.007	−0.003
	0.352	0.485	0.325	0.571	0.957	0.985
KgM	0.023	0.009	0.046	−0.102	0.054	0.042
	0.867	0.951	0.741	0.458	0.693	0.760
RMM	−0.247	0.305	0.289	0.013	−0.118	−0.132
	0.069	0.024*	0.032*	0.923	0.392	0.338
VFR	0.193	−0.084	−0.101	−0.068	−0.246	−0.237
	0.158	0.547	0.465	0.619	0.070	0.082
nVFR	0.211	−0.066	−0.087	−0.014	−0.255	−0.240
	0.123	0.638	0.528	0.918	0.061	0.077

Abbreviations: %FM, fat mass percentage; BMI, body mass index; BR, breathing rate; FuzzEn_PRQ_, fuzzy entropy of PRQ; kgM, muscle kilograms; mPRQ, mean of the PRQ; nVFR, normalized visceral fat rate; RMM, relative muscle mass; RMSSD_BB_, root mean square of successive BB interval differences; SampEn_PRQ_, sample entropy of PRQ; SDBB, standard deviation of BB intervals; VFR, visceral fat rate.

* *p* < 0.05 = Correlation significance level obtained with † Spearman's rho and ‡ Pearson's r.

Among university men, BMI was significantly associated with several BRV‐PRQ measures. Specifically, BMI showed moderate negative correlations with SampEn_PRQ_ and FuzzEn_PRQ_, indicating that higher BMI is associated with lower entropy. Conversely, BMI was positively correlated with SDBB, RMSSD_BB_, and mPRQ, with SDBB showing a moderate positive correlation, suggesting that higher BMI is associated with greater variability in breath‐to‐breath intervals and overall PRQ.

Similarly, %FM in university men exhibited moderate negative correlations with SampEn_PRQ_ and FuzzEn_PRQ_, reinforcing the pattern that higher body fat percentage is linked to lower entropy measures of cardiorespiratory modulation. Additionally, %FM showed a weak positive correlation with SDBB, indicating a relationship between higher body fat and increased variability in breath‐to‐breath intervals.

KgM in university men demonstrated a weak negative correlation with BR, suggesting that more muscle mass is associated with lower breathing rates. Moderate positive correlations were found between KgM and both SDBB and RMSSD_BB_, and RMM in women showed a weak positive association with SDBB and RMSSD_BB_, indicating that greater muscle mass is related to increased variability in breath‐to‐breath intervals.

VFR in university men also showed significant correlations. Moderate negative correlations with SampEn_PRQ_ and FuzzEn_PRQ_ were observed, indicating that higher visceral fat is associated with lower entropy measures. Positive correlations with SDBB, RMSSD_BB_, and mPRQ were also noted, with SDBB and mPRQ showing moderate positive correlations, suggesting that higher visceral fat is linked to increased variability in breath‐to‐breath intervals and overall PRQ.

Lastly, nVFR in university men followed a similar pattern, with moderate negative correlations with SampEn_PRQ_ and FuzzEn_PRQ_, and weak positive correlations with SDBB and mPRQ. This indicates that normalized visceral fat is associated with lower entropy measures but higher variability in breath‐to‐breath intervals and overall PRQ.

In contrast, few significant correlations were found between body composition and respiratory signals in university women, highlighting a clear difference in the relationship between these measures and body composition between sexes. For detailed numerical values and exact correlation coefficients, refer to Table 4.

### 3.1 Regression analysis of fat mass percentage and vagally mediated HRV indices

**FIGURE 2:**
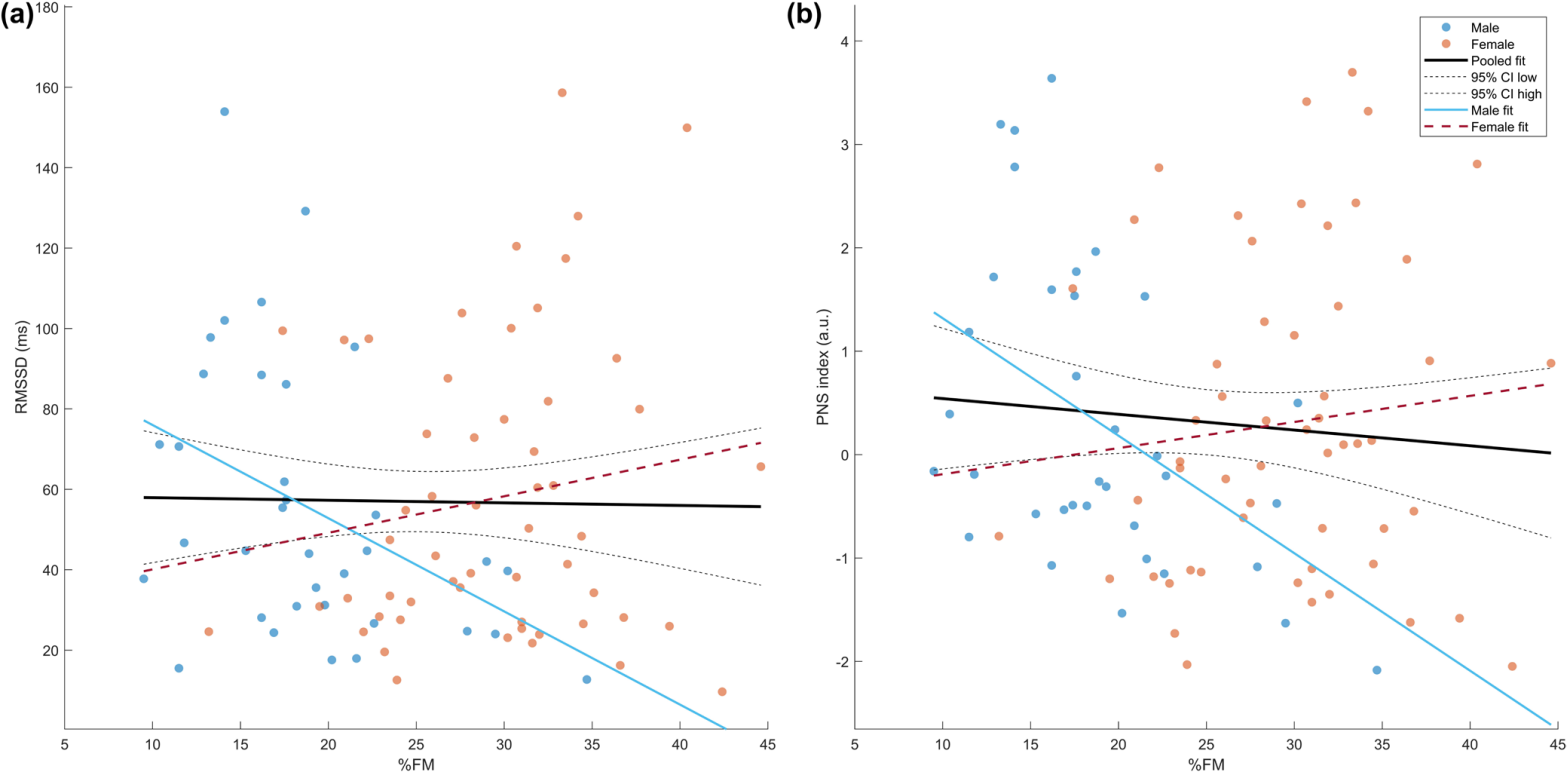
Bivariate associations between fat mass percentage and vagally mediated HRV indices by sex. Scatterplots of fat mass percentage (%FM) versus (a) RMSSD (ms) and (b) PNS index (a.u.) in men (blue) and women (orange). Points represent individual participants. In men, %FM was inversely correlated with RMSSD (Spearman's *rho* = −0.437, *p* = 0.009) and PNS index (Spearman's *rho* = −0.455, *p* = 0.006), whereas no significant correlations were observed in women (RMSSD: *rho* = 0.118, *p* = 0.393; PNS index: *r* = 0.113, *p* = 0.410). The solid black line shows the pooled simple linear regression of each HRV index on %FM, with black dotted lines indicating its 95% confidence interval. The solid blue and dashed red lines depict sex‐specific simple linear fits for men and women, respectively. These regressions are unadjusted; fully adjusted multiple regression models with standardized variables (including age, breathing rate, and total physical activity as covariates) are reported in Table 5.

In pooled multiple robust linear regression models adjusting for sex, age, breathing rate, and total METs, %FM was not significantly associated with RMSSD, HF (n.u.), or the PNS index (all FDR‐adjusted *q*‐values = 0.81; Table 5). However, formal interaction tests indicated that sex significantly moderated the association between %FM and vagally mediated HRV indices. For RMSSD, the %FM × sex interaction was significant (overall model: *F* (6,83) = 3.02, *p* = 0.010; interaction term *p* = 0.011). In sex‐stratified models (Table 5), higher %FM was associated with lower RMSSD in men (β_std_ = −0.48, 95% CI −0.92 to −0.04, *p* = 0.034), whereas no significant association was observed in women (β_std_ = 0.23, 95% CI −0.13 to 0.59, *p* = 0.198; Table 5, Figure 2). A similar but stronger pattern emerged for the PNS index: the %FM × sex interaction was significant (*F* (6, 83) = 3.50, *p* = 0.0039), and in men higher %FM was associated with a marked reduction in PNS modulation (β_std_ = −0.64, 95% CI −1.10 to −0.18, *p* = 0.008, *q*‐value = 0.025), whereas the association in women was small and nonsignificant (β_std_ = 0.20, 95% CI −0.16 to 0.56, *p* = 0.271; Table 5, Figure 2). In contrast, the relationships between %FM and HF (n.u.) did not reach statistical significance in either sex after correction for multiple testing (men: β_std_ = −0.41, *p* = 0.121, *q*‐value = 0.121; women: β_std_ = 0.29, *p* = 0.059, *q*‐value = 0.177; Table 5). In these interaction models, younger age and higher physical activity levels (METs) were also independently associated with higher PNS index, whereas breathing rate was not a significant predictor.

**TABLE 5:** Multiple linear regression models relating fat mass percentage (%FM) to primary vagally mediated HRV indices, adjusted for age, breathing rate, and physical activity (METs), in the full sample and stratified by sex.

Outcome	Sex	β_std_(%FM)	95% CI for β_std_	*p*‐value	*q*‐value (FDR)
RMSSD (ms)	All	−0.05	−0.33 to 0.23	0.715	0.813
HF (n.u.)	All	0.03	−0.24 to 0.31	0.813	0.813
PNS index	All	−0.11	−0.41 to 0.18	0.441	0.813
RMSSD (ms)	Men	−0.48	−0.92 to −0.04	0.034	0.051
HF (n.u.)	Men	−0.41	−0.93 to 0.11	0.121	0.121
PNS index	Men	−0.64	−1.10 to –0.18	0.008*	0.025
RMSSD (ms)	Women	0.23	−0.13 to 0.59	0.198	0.271
HF (n.u.)	Women	0.29	−0.01 to 0.60	0.059	0.177
PNS index	Women	0.20	−0.16 to 0.56	0.271	0.271

*Note*: All models are multiple linear regressions with standardized variables (*z*‐scores). Each model includes %FM as the primary predictor and is adjusted for age, breathing rate (BR), and total physical activity (METs); pooled models additionally adjust for sex. RMSSD, HF (n.u.), and the PNS index were prespecified as vagally mediated HRV indices and treated as primary autonomic outcomes. *q*‐value (FDR): *p*‐value adjusted for multiple testing using the Benjamini–Hochberg false discovery rate procedure within each block of models (all, men, and women). An asterisk (*) denotes significant associations after FDR correction (*q* < 0.05).

Abbreviations: β_std_: standardized regression coefficient; CI, confidence interval; HF (n.u.), high‐frequency power in normalized units; PNS index, parasympathetic nervous system index.

## 4 DISCUSSION

This study identified three main physiological findings: first, respiratory rate was higher in women; this may relate to sex differences in airway caliber and lung volume (LoMauro & Aliverti, 2018). Nevertheless, men exhibited greater respiratory variability than women, as evidenced by higher SDBB and RMSSD_BB_ values. This difference may be partly attributable to testosterone‐related bronchial relaxation (LoMauro & Aliverti, 2021), the larger luminal areas of central and large airways in men (LoMauro & Aliverti, 2018), and their higher high‐frequency systolic blood pressure variability compared with women (Shafiq et al., 2023). Second, body fat and composition were positively associated with sympathetic modulation and negatively associated with parasympathetic modulation, with stronger associations in men; importantly, multivariable regression models confirmed that sex moderates these adiposity–autonomic relationships, indicating that the stronger male associations are not explained solely by covariate effects. In the adjusted models, the association was most consistently captured by the composite PNS index, whereas RMSSD and HF showed directionally consistent effects but did not remain significant in all models after correction. This pattern is consistent with the PNS index being a composite vagal marker (mean RR, RMSSD, and SD1), which may be more robust to noise/collinearity in multivariable models than single indices considered separately. Third, in men, the PRQ, a parameter capturing the complex state of cardiorespiratory interactions, showed significant associations with body composition, particularly entropy measures of the PRQ time series, highlighting the intricate balance of the cardiorespiratory autonomic regulation.

To our knowledge, this is the first study to analyze the association between body composition and HRV, in conjunction with BRV and PRQ, in a healthy young population. In contrast, other studies typically focus on populations with specific diseases (Escutia‐Reyes et al., 2021; Plaza‐Florido et al., 2019) or athletes (Kothari et al., 2023).

### 4.1 BMI and cardiorespiratory autonomic regulation (HRV/BRV/PRQ)

According to the BMI classification, most participants were of normal weight (60.0%), 36.7% were overweight, and only one participant (1.1%) met the BMI criteria for obesity. When classified by body‐fat percentage, 10% of the total sample had obesity (men: 11.4%; women: 9.1%). Analysis revealed that only in men, BMI is negatively associated with parasympathetic modulation of HRV, including the PNS index, pNN50, RMSSD, and HFnu, which are also linked to the respiratory cycle. Conversely, BMI showed positive associations with sympathetic indices, such as the SNS index, as well as with SDBB and RMSSD_BB_. Additionally, negative correlations were observed between BMI and entropy indices for both cardiac (SampEn) and PRQ (SampEn and FuzzEn) measures. These findings are consistent, in this sample, with group‐level associations between higher BMI and less favorable autonomic profiles, including lower vagally mediated HRV indices and reduced complexity, aligning with previous studies that reported negative associations between BMI and global HRV (SDRR) (Chang et al., 2021) and parasympathetic modulation (RMSSD and HF) (Monteze et al., 2015).

The observation that the human heart rate is approximately four times that of the respiratory rate has been recognized for centuries. Previous evidence highlighted the significant potential of the PRQ for understanding human physiology, particularly the importance of the PRQ value of 4, which indicates a 4:1 ratio of cardiac activity to respiration (Scholkmann & Wolf, 2019). In men, the mean PRQ (mPRQ) was positively correlated with BMI, VFR, and normalized VFR. Physiologically, this suggests that individuals with higher BMI and visceral fat rates may be associated with higher mPRQ, reflecting an increased heart rate relative to their respiration rate. This elevation in mPRQ suggests a shift towards greater sympathetic dominance and reduced parasympathetic modulation, indicating a less adaptive and more strained autonomic regulation.

The positive correlation between mPRQ and BMI suggests that excess body weight, particularly visceral fat, is associated with a higher heart rate for a given respiratory rate, a sign of increased cardiovascular strain. Similarly, associations with VFR and normalized VFR indicate that visceral fat, which is more metabolically active and associated with higher inflammation, further exacerbates this imbalance.

These positive associations suggest that the cardiorespiratory state could elicit autonomic adaptations across various stressors, including those associated with excess weight, particularly visceral adipose tissue (VAT) (Habib et al., 2024; Triggiani et al., 2019). This cardiorespiratory adaptation has also been observed in women with severe preeclampsia (Pichardo‐Carmona et al., 2023) and individuals with autonomic dysfunction (Scholkmann & Wolf, 2019). It is crucial to note that reducing fat mass (FM) and visceral fat mass (VFM) can enhance parasympathetic modulation and aerobic capacity (Phoemsapthawee et al., 2019), highlighting the importance of maintaining a healthy body composition for optimal autonomic and cardiovascular health.

### 4.2 Adiposity markers (%FM, VFR, and nVFR) and autonomic balance and complexity (HRV/PRQ)

Although the incidence of obesity diagnosed by %FM in men is relatively low (11.4%), this study found %FM to be negatively associated with cardiac parasympathetic modulation (PNS index, pNN50, RMSSD, and HFnu) and overall heart rate variability or SDRR, while positively associated with cardiac sympathetic modulation (SNS index and LF). The literature presents mixed results regarding the association between FM and sympathetic modulation, with some studies indicating a positive association (Cvijetic et al., 2022) and others a negative one (Cvijetic et al., 2023; Millis et al., 2010). Nonetheless, there is consensus that FM influences HRV.

Similarly, our findings indicate that VFR, as measured by BIA, was negatively associated with parasympathetic modulation indices (PNS index, pNN50) related to the respiratory cycle (RMSSD, HF) and overall heart rate variability (SDRR) only in men. Conversely, VFR is positively associated with sympathetic modulation indices (SNS index and LF) in men as well. These results are consistent with previous research, which found that in healthy overweight men, visceral fat (VF) is negatively associated with parasympathetic fluctuations (HF) and positively with sympathetic fluctuations (LF) (Chintala et al., 2015). Additionally, higher levels of VAT have been shown to decrease HRV (Triggiani et al., 2019). Studies on the impact of age on parasympathetic modulation have yielded contradictory results, with some reporting lower parasympathetic modulation in younger individuals (Windham et al., 2012) and others noting a decrease with aging (Jasrotia et al., 2019). Despite these discrepancies, there is consensus that VF plays a crucial role in the deterioration of parasympathetic modulation. This implies that individuals with a high visceral fat percentage from an early age may experience premature aging of the autonomic nervous system (ANS).

In this study, nVFR was found to be negatively associated with PNS index, pNN50, RMSSD, and HFnu, and positively associated with sympathetic modulation indices (SNS index and LF) in men, which aligns with findings from a study on women breast cancer survivors (Escutia‐Reyes et al., 2021).

A key aspect of this research is its novel correlation of nonlinear cardiac and respiratory parameters with body composition. In men, BMI, %FM, VFR, and nVFR were all negatively associated with cardiac entropy (SampEn) and entropy of the PRQ (SampEn and FuzzEn). This suggests that excess weight, particularly visceral fat, can compromise the cardiorespiratory system, especially in men. Previous studies have shown that complexity decreases with age (Bhogal & Mani, 2017), leading to an imbalance in cardiorespiratory control, which can increase the risk of additional pathologies or exacerbate existing conditions, as seen in patients with coronary artery disease (Chen et al., 2017) and diabetes (Khandoker et al., 2017; Li et al., 2015).

### 4.3 Muscle mass (kgM) and cardiorespiratory indices (HRV/BRV)

Studies in healthy populations have found that KgM is associated with increased parasympathetic (HF) modulation (Cvijetic et al., 2023; Jasrotia et al., 2019), possibly because the study populations engaged in regular physical activity or had better body composition. In this sense, it is essential to mention that our population has an average muscle mass for its age (Cruz‐Jentoft et al., 2019) (Men = 53.3 ± 5.2; Women = 39.5 ± 2.8, *p* = < 0.05), associated with the fact that the majority perform physical activity from moderate (40%) to vigorous (41.1%), with the practice of vigorous physical activity (VPA) being more common in men than in women (*p* = 0.05; 57.1% and 30.9%, respectively).

However, when correlating cardiorespiratory indices with kgM, we found that in men, kgM was negatively associated with cardiac parasympathetic indices (pNN50 and HF), tended to be inversely associated with BR, and was positively associated with respiratory variability (SDBB; RMSSD_BB_). This suggests that, in men, greater muscle mass was associated with lower vagally mediated HRV indices but was accompanied by higher respiratory variability. Although muscle mass is usually considered protective, kgM in this study represents absolute muscle mass and is strongly related to total body size. Therefore, higher kgM in men may reflect a larger body habitus that co‐varies with adiposity rather than greater relative muscularity. In line with this context, Habib et al. found in healthy adult males that visceral fat rating was inversely associated with vagally mediated HRV indices and that a muscle‐to‐visceral‐fat ratio also related negatively to HRV, highlighting that muscle measures embedded in visceral adiposity variation may not behave as purely protective markers (Habib et al., 2024). Moreover, vagally mediated HRV indices (e.g., RMSSD and HF) are highly heart‐rate dependent, and part of the kgM–HRV slope may therefore reflect heart‐rate/body‐size scaling. Consistently, comprehensive reviews indicate that diminished HRV is associated with higher fat percentage and a lower percentage of muscle mass (Sánchez et al., 2015), implying that the protective component is relative muscularity rather than absolute kgM. Similar reductions in parasympathetic correlations (pNN50 and RMSSD) (de Freitas et al., 2018) and autonomic modulation (SDNN, HF, and LF) (Zheng et al., 2024) have been reported in older adults with sarcopenia.

To our knowledge, this is the first study correlating BRV with body composition; thus, we found no prior studies for direct comparison. However, specific voluntary breathing exercises are known to modulate the ANS by increasing PNS activation and decreasing SNS modulation (Inthachai et al., 2020; Jerath et al., 2006). Likewise, in the literature, the effect of physical activity on autonomic functioning has been widely described (Marasingha‐Arachchige et al., 2022), with moderate‐to‐vigorous physical activity (Alansare et al., 2022), increasing parasympathetic modulation (RMSSD) and overall HRV (SDNN). Also, adopting exercise in people with a disease (Caruso et al., 2015; El‐Malahi et al., 2024; Su et al., 2022) improves autonomic modulation (SDNN and RMSSD).

Compared to men, kgM in women had a statistically significant negative association with LF (sympathetic) and a positive association with HF (parasympathetic). This suggests that greater muscle mass could be beneficial for women. Other studies have shown that muscle mass is associated with improved HRV (Cvijetic et al., 2023). However, the physiological importance of LF in regulating cardiac sympathetic tone remains unclear in the literature (Goldstein et al., 2011).

### 4.4 Limitations and future directions

Our study presents several limitations that should be acknowledged. First, our sample size was relatively modest, consisting of 90 clinically healthy Mexican university students from a single institution, which limits the generalizability of our findings to other age groups, settings, and clinical populations. In addition, the BMI and adiposity range was relatively narrow, with very few participants meeting BMI‐based obesity criteria, which limits extrapolation of these associations to obese or metabolically compromised populations. Together, these factors support interpreting the present results as exploratory and hypothesis‐generating. Second, our study employs a cross‐sectional design, which precludes causal inference or determination of the direction of the associations.

Third, body composition was assessed using BIA, which is not considered a gold‐standard method and provides only indirect estimates of total and visceral fat. Hydration status, individual body composition, pre‐examination conditions, and body position can influence its accuracy. In particular, hydration status was not standardized or monitored, which may have introduced additional variability in %FM and visceral fat estimates. The visceral fat indices (VFR and nVFR) used in our analyses should be interpreted as approximate, device‐specific proxies rather than precise measures of central adiposity. In addition, we did not collect direct anthropometric markers of central adiposity (e.g., waist circumference or waist‐to‐hip ratio), and our assessment of fat distribution relied exclusively on BIA‐derived indices (%FM, VFR, and nVFR) and BMI. Future studies should incorporate gold‐standard techniques, such as dual‐energy X‐ray absorptiometry (DEXA) and, preferably, imaging‐based measures in combination with anthropometric indices to provide a more robust characterization of body fat distribution.

Additionally, current smoking was more frequent in men and may have influenced autonomic and respiratory indices; therefore, sex‐related respiratory findings should be interpreted cautiously. Baseline BR and BRV sex differences are now shown in Table 2 and Figure S1.

Fourth, we did not control covariables such as the female menstrual cycle, which may have introduced additional variability and should be considered in future studies. Finally, a controlled breathing protocol was not implemented, which might have influenced spontaneous baroreflex sensitivity and HRV measurements (Guzik et al., 2007), particularly in women.

## 5 CONCLUSION

This study shows that, even in clinically healthy young adults with a predominantly nonobese profile, body composition relates to autonomic cardiorespiratory regulation in a sex‐dependent manner. First, clear respiratory sex differences emerged: women exhibited higher resting breathing rate, whereas men showed lower breathing rate but greater breath‐to‐breath variability (higher SDBB and RMSSD_BB_). Second, adiposity markers (BMI, %FM, VFR, and nVFR) were associated with lower vagally mediated HRV in men, with the strongest evidence in adjusted analyses for the composite PNS index; RMSSD, pNN50, and HF showed directionally consistent associations but were less robust across models, and with higher sympathetic‐related indices (SNS, LF) in men. Still, these associations were weak or absent in women. Third, the pulse–respiration quotient (PRQ) and its entropy measures were significantly associated with body composition in men, suggesting a link with cardiorespiratory coupling. In this sample, elevated body fat, especially visceral fat, was associated with cardiorespiratory profiles characterized by lower physiological complexity and adaptability.

Importantly, multivariable models were adjusted for sex, age, breathing rate, and habitual physical activity (METs/week). Overall, these findings suggest that subtle adiposity–autonomic associations are already detectable in early adulthood and are sex‐moderated (more evident in men), and they coincide with clear sex differences in breathing patterning (lower breathing rate but higher breath‐to‐breath variability in men) and with adiposity‐related associations in PRQ‐derived indices.

## FUNDING INFORMATION

This research was supported by the Secretaría de Ciencias, Humanidades, Tecnología e Innovación (SECIHTI, CVU: 998759). The funding agency had no role in study design, data collection, analysis, or manuscript writing.

## CONFLICT OF INTEREST STATEMENT

The authors declare no conflict of interest regarding the preparation of the present manuscript.

## ETHICS STATEMENT

This study was conducted in accordance with the principles of the Declaration of Helsinki. All participants provided written informed consent prior to participation. The study protocol was approved by the Research Ethics Committee of the School of Medicine, Autonomous University of the State of Mexico (approval/registry number: 005.2023).

## Supporting information


Figure S1.

## Data Availability

The anonymized dataset and the derived autonomic/respiratory indices supporting the findings of this study are publicly available in the Universidad Autónoma Metropolitana–Unidad Lerma institutional repository (XOGI) at http://hdl.handle.net/20.500.12222/439 and https://xogi.ler.uam.mx/items/bc1de4dc‐3e69‐418e‐98ff‐f066bf7c06fa.
