# Supplementary material for: Associations between body composition and autonomic cardiorespiratory modulation in young adults
Source: Physiol Rep. 2026 Feb 9;14(3):e70760. doi: 10.14814/phy2.70760 (PMC12885932; doi:10.14814/phy2.70760)
Supplement: Supplementary file 1 — Figure S1. [file PHY2-14-e70760-s001.docx]

**Supplementary material S1**

**
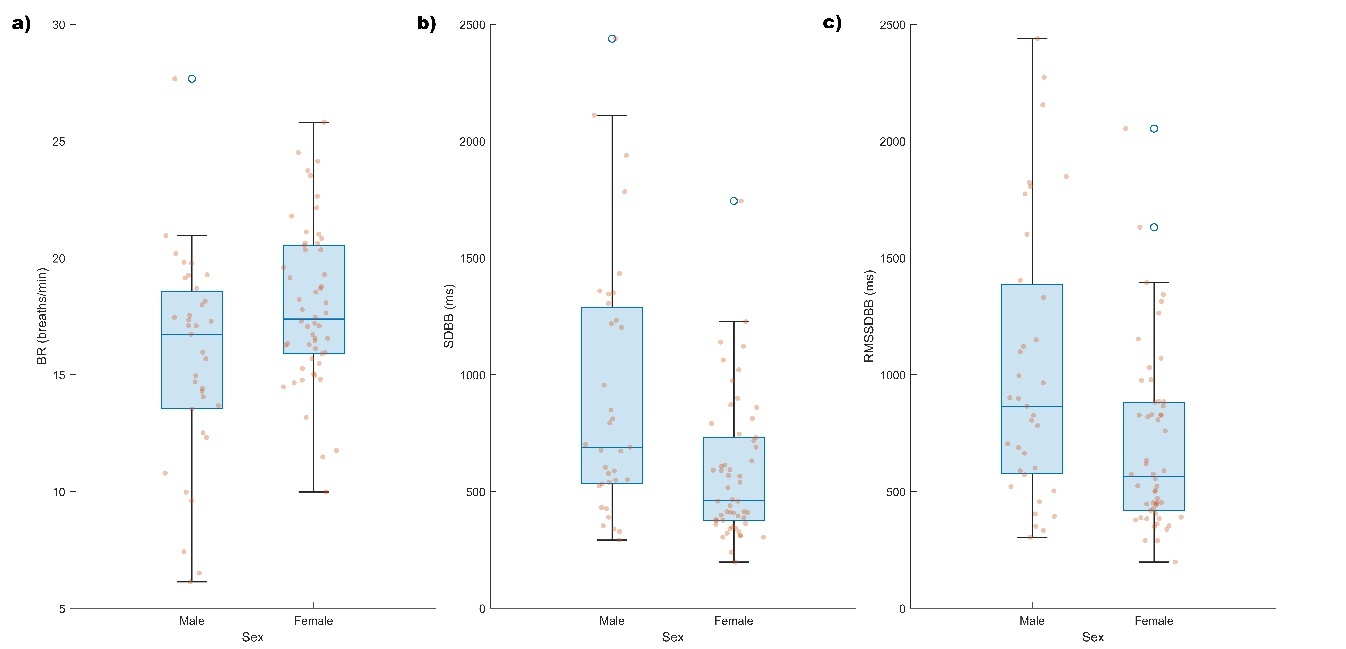
**

Fig. S1. Sex differences in respiratory indices. (a) Breathing rate (BR, breaths/min), (b) standard deviation of breath-to-breath intervals (SDBB, ms), and (c) root mean square of successive differences of breath-to-breath intervals (RMSSDBB, ms) in males (n = 35) and females (n = 55). Boxes indicate the median and interquartile range (IQR), whiskers extend to 1.5×IQR, jittered points represent individual participants, and open circles denote outliers. Between-sex differences were assessed using the same statistical approach described in the Methods; significant comparisons are indicated in the Results/Table (*p < 0.05).
